# 5,6-Dimethyl­pyrazine-2,3-dicarbonitrile

**DOI:** 10.1107/S1600536812043474

**Published:** 2012-10-27

**Authors:** Ghasem Rezanejade Bardajee, Alan J. Lough, Mitchell A. Winnik

**Affiliations:** a Department of Chemistry, Payame Noor University, PO Box 19395-3697, Tehran, Iran; b Department of Chemistry, University of Toronto, 80 St Geroge St, Toronto, Ontario, Canada M5S 3H6

## Abstract

The asymmetric unit of the title compound, C_8_H_6_N_4_, contains two almost planar independent mol­ecules (r.m.s. deviations = 0.026 and 0.030 Å). The crystal studied was a non-merohedral twin with the components in a 0.513 (2):0.487 (2) ratio.

## Related literature

For applications of pyrazine compounds and their derivatives, see: He *et al.* (2003 ▶); Yadav *et al.* (2008 ▶). For the synthesis, see: Bardajee *et al.* (2012 ▶). For related structures, see: Hökelek *et al.* (2009 ▶); Donzello *et al.* (2004 ▶); Cristiano *et al.* (2007 ▶).
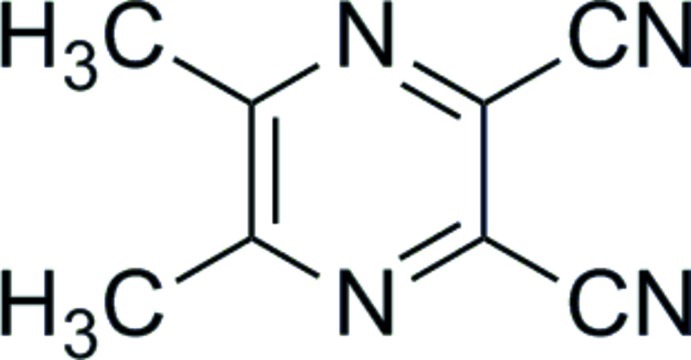


## Experimental

### 

#### Crystal data

C_8_H_6_N_4_
*M* *_r_* = 158.17
Monoclinic, 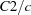

*a* = 24.183 (2) Å
*b* = 9.210 (1) Å
*c* = 18.761 (2) Å
β = 130.151 (2)°
*V* = 3193.8 (6) Å^3^
*Z* = 16
Mo *K*α radiation
μ = 0.09 mm^−1^
*T* = 150 K
0.28 × 0.22 × 0.18 mm

#### Data collection

Bruker Kappa APEXII DUO CCD diffractometer
Absorption correction: multi-scan (*SADABS*; Bruker, 2007 ▶) *T*_min_ = 0.711, *T*_max_ = 0.746
7543 measured reflections
3650 independent reflections
2927 reflections with *I* > 2σ(*I*)
*R*_int_ = 0.034

#### Refinement

*R*[*F* ^2^ > 2σ(*F* ^2^)] = 0.039
*wR*(*F* ^2^) = 0.110
*S* = 1.05
3650 reflections
222 parameters
H-atom parameters constrained
Δρ_max_ = 0.21 e Å^−3^
Δρ_min_ = −0.21 e Å^−3^

### 

Data collection: *APEX2* (Bruker, 2007 ▶); cell refinement: *SAINT* (Bruker, 2007 ▶); data reduction: *SAINT*; program(s) used to solve structure: *SHELXS97* (Sheldrick, 2008 ▶); program(s) used to refine structure: *SHELXL97* (Sheldrick, 2008 ▶); molecular graphics: *PLATON* (Spek, 2009 ▶); software used to prepare material for publication: *SHELXTL* (Sheldrick, 2008 ▶).

## Supplementary Material

Crystal structure: contains datablock(s) global, I. DOI: 10.1107/S1600536812043474/hb6967sup1.cif

Structure factors: contains datablock(s) I. DOI: 10.1107/S1600536812043474/hb6967Isup2.hkl

Supplementary material file. DOI: 10.1107/S1600536812043474/hb6967Isup3.cml

Additional supplementary materials:  crystallographic information; 3D view; checkCIF report

## Figures and Tables

**Fig. 1.**
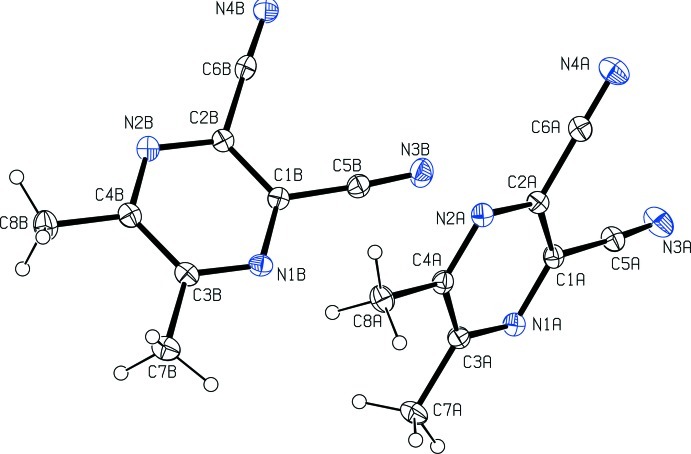
The asymmetric unit of the title compound.

## supplementary crystallographic information

### Comment

Pyrazine is a nitrogen containing heterocycle and is a major scaffold for other heterocycles such as pyridopyrazines and quinoxalines. These compounds have received considerable attention in the pharmaceutical industry because of their interesting therapeutic properties (He *et al.*, 2003; Yadav *et al.*, 2008). Herein, we report the crystal structure of the title compound (I).

The asymmetric unit of (I) contains two independent molecules (A and B) (Fig. 1). In (I) the bond distances are similar to the equivalent distances in 5,6-diphenylpyrazine-2,3-dicarbonitrile (Hökelek *et al.*, 2009), 5,6-bis(2-pyridyl)-2,3-pyrazinedicarbonitrile (Donzello *et al.*, 2004) and 5,6-bis(4-methoxyphenyl)-2,3-pyrazinedicarbonitrile (Cristiano *et al.*, 2007).

### Experimental

The title compound was synthesized from the reaction of 2,3-diaminomaleonitrile and biacetyl in the presence a heterogeneous catalyst based on copper bearing salen Schiff base ligands covalently anchored into SBA-15 in water (Bardajee *et al.* 2012). Colourless blocks were grown from a solution of the title compound in ethanol.

### Refinement

Hydrogen atoms were placed in calculated positions with C—H distances of 0.98 Å and were included in the refinement in a riding-model approximation with *U*_iso_(H) = 1.5*U*_eq_(C). The crystal studied was a non-merohedral twin with twin law -1 0 0, 0 - 1 0, 1 0 1 and with the components in a ratio of 0.513 (2):0.487 (2).

### Crystal data

**Table:**

C_8_H_6_N_4_	*F*(000) = 1312
*M**_r_* = 158.17	*D*_x_ = 1.316 Mg m^−^^3^
Monoclinic, *C*2/*c*	Mo *K*α radiation, λ = 0.71073 Å
Hall symbol: -C 2yc	Cell parameters from 3553 reflections
*a* = 24.183 (2) Å	θ = 2.5–27.5°
*b* = 9.210 (1) Å	µ = 0.09 mm^−^^1^
*c* = 18.761 (2) Å	*T* = 150 K
β = 130.151 (2)°	Block, colourless
*V* = 3193.8 (6) Å^3^	0.28 × 0.22 × 0.18 mm
*Z* = 16	

### Data collection

**Table:**

Bruker Kappa APEXII DUO CCD diffractometer	3650 independent reflections
Radiation source: fine-focus sealed tube	2927 reflections with *I* > 2σ(*I*)
Bruker Triumph monochromator	*R*_int_ = 0.034
φ and ω scans	θ_max_ = 27.5°, θ_min_ = 2.2°
Absorption correction: multi-scan (*SADABS*; Bruker, 2007)	*h* = −31→31
*T*_min_ = 0.711, *T*_max_ = 0.746	*k* = −11→11
7543 measured reflections	*l* = −23→24

### Refinement

**Table:**

Refinement on *F*^2^	Primary atom site location: structure-invariant direct methods
Least-squares matrix: full	Secondary atom site location: difference Fourier map
*R*[*F*^2^ > 2σ(*F*^2^)] = 0.039	Hydrogen site location: inferred from neighbouring sites
*wR*(*F*^2^) = 0.110	H-atom parameters constrained
*S* = 1.05	*w* = 1/[σ^2^(*F*_o_^2^) + (0.057*P*)^2^ + 0.5454*P*] where *P* = (*F*_o_^2^ + 2*F*_c_^2^)/3
3650 reflections	(Δ/σ)_max_ = 0.001
222 parameters	Δρ_max_ = 0.21 e Å^−^^3^
0 restraints	Δρ_min_ = −0.21 e Å^−^^3^

### Special details

**Table:**

Geometry. All e.s.d.'s (except the e.s.d. in the dihedral angle between two l.s. planes) are estimated using the full covariance matrix. The cell e.s.d.'s are taken into account individually in the estimation of e.s.d.'s in distances, angles and torsion angles; correlations between e.s.d.'s in cell parameters are only used when they are defined by crystal symmetry. An approximate (isotropic) treatment of cell e.s.d.'s is used for estimating e.s.d.'s involving l.s. planes.
Refinement. Refinement of *F*^2^ against ALL reflections. The weighted *R*-factor *wR* and goodness of fit *S* are based on *F*^2^, conventional *R*-factors *R* are based on *F*, with *F* set to zero for negative *F*^2^. The threshold expression of *F*^2^ > σ(*F*^2^) is used only for calculating *R*-factors(gt) *etc*. and is not relevant to the choice of reflections for refinement. *R*-factors based on *F*^2^ are statistically about twice as large as those based on *F*, and *R*- factors based on ALL data will be even larger.

### Fractional atomic coordinates and isotropic or equivalent isotropic displacement parameters (Å^2^)

**Table:**

	*x*	*y*	*z*	*U*_iso_*/*U*_eq_	
N1A	0.04856 (10)	0.48725 (16)	0.69738 (13)	0.0248 (4)	
N2A	0.19891 (9)	0.49247 (15)	0.79770 (12)	0.0243 (4)	
N3A	0.02419 (9)	0.11627 (19)	0.68396 (15)	0.0406 (4)	
N4A	0.23018 (9)	0.12325 (19)	0.81987 (14)	0.0376 (4)	
C1A	0.08817 (10)	0.3648 (2)	0.72638 (13)	0.0236 (4)	
C2A	0.16196 (10)	0.3674 (2)	0.77523 (12)	0.0227 (4)	
C3A	0.08416 (11)	0.6117 (2)	0.71857 (12)	0.0249 (4)	
C4A	0.16036 (11)	0.6147 (2)	0.76943 (14)	0.0245 (4)	
C5A	0.05086 (12)	0.2266 (2)	0.70240 (16)	0.0275 (5)	
C6A	0.20185 (12)	0.2326 (2)	0.80236 (17)	0.0269 (5)	
C7A	0.04121 (14)	0.7493 (2)	0.68703 (18)	0.0338 (6)	
H7AA	−0.0082	0.7271	0.6615	0.051*	
H7AB	0.0398	0.7954	0.6388	0.051*	
H7AC	0.0639	0.8155	0.7402	0.051*	
C8A	0.19974 (14)	0.7552 (2)	0.79508 (18)	0.0312 (5)	
H8AA	0.2503	0.7364	0.8246	0.047*	
H8AB	0.1977	0.8079	0.8387	0.047*	
H8AC	0.1771	0.8138	0.7388	0.047*	
N1B	0.09889 (10)	0.74478 (15)	0.54586 (13)	0.0259 (4)	
N2B	0.14892 (10)	0.73602 (17)	0.44671 (13)	0.0272 (4)	
N3B	0.07877 (10)	0.3751 (2)	0.55725 (13)	0.0378 (4)	
N4B	0.15302 (10)	0.36510 (18)	0.42592 (13)	0.0367 (4)	
C1B	0.10706 (10)	0.6194 (2)	0.51766 (13)	0.0234 (4)	
C2B	0.13235 (10)	0.6149 (2)	0.46911 (13)	0.0234 (4)	
C3B	0.11532 (10)	0.8655 (2)	0.52460 (13)	0.0258 (4)	
C4B	0.14069 (10)	0.8614 (2)	0.47436 (13)	0.0268 (4)	
C5B	0.09057 (12)	0.4855 (2)	0.54051 (15)	0.0276 (5)	
C6B	0.14345 (12)	0.4770 (3)	0.44367 (16)	0.0277 (5)	
C7B	0.10652 (13)	1.0064 (3)	0.55575 (18)	0.0347 (6)	
H7BA	0.0831	0.9892	0.5823	0.052*	
H7BB	0.0766	1.0721	0.5023	0.052*	
H7BC	0.1541	1.0504	0.6031	0.052*	
C8B	0.15737 (15)	0.9966 (3)	0.44719 (19)	0.0385 (6)	
H8BA	0.1780	0.9705	0.4180	0.058*	
H8BB	0.1922	1.0555	0.5029	0.058*	
H8BC	0.1128	1.0523	0.4029	0.058*	

### Atomic displacement parameters (Å^2^)

**Table:**

	*U*^11^	*U*^22^	*U*^33^	*U*^12^	*U*^13^	*U*^23^
N1A	0.0244 (9)	0.0262 (8)	0.0213 (8)	−0.0012 (6)	0.0135 (8)	−0.0013 (6)
N2A	0.0252 (9)	0.0241 (9)	0.0209 (8)	−0.0027 (6)	0.0136 (8)	−0.0024 (6)
N3A	0.0297 (9)	0.0301 (8)	0.0433 (9)	−0.0022 (7)	0.0150 (8)	0.0007 (8)
N4A	0.0269 (8)	0.0308 (8)	0.0386 (9)	0.0008 (7)	0.0135 (8)	−0.0011 (7)
C1A	0.0265 (9)	0.0230 (10)	0.0198 (8)	−0.0027 (8)	0.0142 (8)	−0.0011 (7)
C2A	0.0217 (9)	0.0237 (9)	0.0183 (8)	−0.0001 (7)	0.0109 (7)	−0.0010 (7)
C3A	0.0306 (10)	0.0235 (9)	0.0215 (9)	0.0014 (8)	0.0172 (8)	−0.0013 (7)
C4A	0.0289 (10)	0.0238 (9)	0.0232 (9)	−0.0033 (8)	0.0178 (8)	−0.0027 (7)
C5A	0.0205 (10)	0.0266 (11)	0.0260 (9)	−0.0008 (8)	0.0107 (8)	0.0018 (8)
C6A	0.0220 (10)	0.0256 (11)	0.0265 (10)	−0.0046 (8)	0.0127 (9)	−0.0022 (8)
C7A	0.0375 (13)	0.0221 (12)	0.0402 (13)	0.0047 (8)	0.0242 (12)	0.0000 (8)
C8A	0.0340 (12)	0.0226 (12)	0.0319 (11)	−0.0049 (8)	0.0190 (10)	−0.0034 (7)
N1B	0.0249 (9)	0.0257 (10)	0.0246 (10)	0.0005 (6)	0.0148 (8)	−0.0026 (6)
N2B	0.0265 (10)	0.0275 (9)	0.0250 (9)	−0.0025 (6)	0.0155 (9)	0.0003 (6)
N3B	0.0564 (12)	0.0305 (8)	0.0387 (9)	−0.0069 (8)	0.0362 (9)	−0.0043 (7)
N4B	0.0504 (11)	0.0293 (8)	0.0418 (9)	0.0023 (8)	0.0349 (9)	0.0005 (7)
C1B	0.0215 (9)	0.0252 (10)	0.0201 (8)	−0.0014 (7)	0.0119 (8)	−0.0008 (7)
C2B	0.0213 (9)	0.0246 (10)	0.0198 (8)	−0.0013 (7)	0.0113 (8)	−0.0008 (7)
C3B	0.0187 (9)	0.0250 (10)	0.0226 (9)	−0.0008 (7)	0.0083 (8)	−0.0009 (7)
C4B	0.0216 (9)	0.0274 (10)	0.0231 (9)	−0.0002 (8)	0.0106 (8)	0.0010 (8)
C5B	0.0333 (11)	0.0283 (12)	0.0242 (10)	−0.0009 (8)	0.0198 (9)	−0.0025 (8)
C6B	0.0294 (11)	0.0323 (12)	0.0247 (10)	−0.0021 (9)	0.0190 (9)	0.0015 (8)
C7B	0.0325 (12)	0.0301 (12)	0.0384 (12)	0.0015 (9)	0.0215 (11)	−0.0023 (9)
C8B	0.0439 (14)	0.0301 (13)	0.0432 (14)	−0.0044 (9)	0.0288 (12)	0.0023 (9)

### Geometric parameters (Å, º)

**Table:**

N1A—C3A	1.331 (2)	N1B—C3B	1.326 (3)
N1A—C1A	1.346 (3)	N1B—C1B	1.337 (3)
N2A—C4A	1.334 (2)	N2B—C4B	1.333 (3)
N2A—C2A	1.348 (2)	N2B—C2B	1.341 (3)
N3A—C5A	1.132 (3)	N3B—C5B	1.153 (3)
N4A—C6A	1.141 (3)	N4B—C6B	1.151 (3)
C1A—C2A	1.384 (3)	C1B—C2B	1.387 (3)
C1A—C5A	1.454 (3)	C1B—C5B	1.444 (3)
C2A—C6A	1.448 (3)	C2B—C6B	1.442 (3)
C3A—C4A	1.428 (3)	C3B—C4B	1.418 (3)
C3A—C7A	1.497 (3)	C3B—C7B	1.494 (3)
C4A—C8A	1.491 (3)	C4B—C8B	1.496 (3)
C7A—H7AA	0.9800	C7B—H7BA	0.9800
C7A—H7AB	0.9800	C7B—H7BB	0.9800
C7A—H7AC	0.9800	C7B—H7BC	0.9800
C8A—H8AA	0.9800	C8B—H8BA	0.9800
C8A—H8AB	0.9800	C8B—H8BB	0.9800
C8A—H8AC	0.9800	C8B—H8BC	0.9800
			
C3A—N1A—C1A	116.49 (17)	C3B—N1B—C1B	117.12 (19)
C4A—N2A—C2A	116.36 (17)	C4B—N2B—C2B	116.69 (19)
N1A—C1A—C2A	122.05 (17)	N1B—C1B—C2B	121.73 (17)
N1A—C1A—C5A	118.06 (17)	N1B—C1B—C5B	118.72 (18)
C2A—C1A—C5A	119.86 (17)	C2B—C1B—C5B	119.53 (17)
N2A—C2A—C1A	122.25 (17)	N2B—C2B—C1B	121.89 (17)
N2A—C2A—C6A	117.74 (17)	N2B—C2B—C6B	118.18 (18)
C1A—C2A—C6A	120.00 (17)	C1B—C2B—C6B	119.91 (17)
N1A—C3A—C4A	121.55 (17)	N1B—C3B—C4B	121.32 (17)
N1A—C3A—C7A	117.42 (18)	N1B—C3B—C7B	117.71 (19)
C4A—C3A—C7A	121.03 (18)	C4B—C3B—C7B	120.97 (19)
N2A—C4A—C3A	121.30 (16)	N2B—C4B—C3B	121.25 (18)
N2A—C4A—C8A	117.84 (18)	N2B—C4B—C8B	116.6 (2)
C3A—C4A—C8A	120.83 (17)	C3B—C4B—C8B	122.12 (18)
N3A—C5A—C1A	176.9 (2)	N3B—C5B—C1B	176.8 (2)
N4A—C6A—C2A	176.6 (3)	N4B—C6B—C2B	177.9 (2)
C3A—C7A—H7AA	109.5	C3B—C7B—H7BA	109.5
C3A—C7A—H7AB	109.5	C3B—C7B—H7BB	109.5
H7AA—C7A—H7AB	109.5	H7BA—C7B—H7BB	109.5
C3A—C7A—H7AC	109.5	C3B—C7B—H7BC	109.5
H7AA—C7A—H7AC	109.5	H7BA—C7B—H7BC	109.5
H7AB—C7A—H7AC	109.5	H7BB—C7B—H7BC	109.5
C4A—C8A—H8AA	109.5	C4B—C8B—H8BA	109.5
C4A—C8A—H8AB	109.5	C4B—C8B—H8BB	109.5
H8AA—C8A—H8AB	109.5	H8BA—C8B—H8BB	109.5
C4A—C8A—H8AC	109.5	C4B—C8B—H8BC	109.5
H8AA—C8A—H8AC	109.5	H8BA—C8B—H8BC	109.5
H8AB—C8A—H8AC	109.5	H8BB—C8B—H8BC	109.5
			
C3A—N1A—C1A—C2A	−0.4 (3)	C3B—N1B—C1B—C2B	0.7 (3)
C3A—N1A—C1A—C5A	−178.40 (18)	C3B—N1B—C1B—C5B	179.24 (18)
C4A—N2A—C2A—C1A	−0.7 (3)	C4B—N2B—C2B—C1B	0.7 (3)
C4A—N2A—C2A—C6A	178.1 (2)	C4B—N2B—C2B—C6B	−177.5 (2)
N1A—C1A—C2A—N2A	0.7 (3)	N1B—C1B—C2B—N2B	−1.0 (3)
C5A—C1A—C2A—N2A	178.75 (17)	C5B—C1B—C2B—N2B	−179.54 (17)
N1A—C1A—C2A—C6A	−178.02 (17)	N1B—C1B—C2B—C6B	177.20 (19)
C5A—C1A—C2A—C6A	0.0 (3)	C5B—C1B—C2B—C6B	−1.3 (3)
C1A—N1A—C3A—C4A	0.0 (3)	C1B—N1B—C3B—C4B	−0.2 (3)
C1A—N1A—C3A—C7A	−179.98 (19)	C1B—N1B—C3B—C7B	−179.94 (19)
C2A—N2A—C4A—C3A	0.3 (3)	C2B—N2B—C4B—C3B	−0.3 (3)
C2A—N2A—C4A—C8A	178.52 (19)	C2B—N2B—C4B—C8B	−178.47 (19)
N1A—C3A—C4A—N2A	0.0 (3)	N1B—C3B—C4B—N2B	0.0 (3)
C7A—C3A—C4A—N2A	−179.99 (18)	C7B—C3B—C4B—N2B	179.73 (19)
N1A—C3A—C4A—C8A	−178.13 (18)	N1B—C3B—C4B—C8B	178.10 (19)
C7A—C3A—C4A—C8A	1.8 (3)	C7B—C3B—C4B—C8B	−2.2 (3)
